# Runx2/Osterix and Zinc Uptake Synergize to Orchestrate Osteogenic Differentiation and Citrate Containing Bone Apatite Formation

**DOI:** 10.1002/advs.201700755

**Published:** 2018-01-28

**Authors:** Xuekun Fu, Yunyan Li, Tongling Huang, Zhiwu Yu, Kun Ma, Meng Yang, Qingli Liu, Haobo Pan, Huaiyu Wang, Junfeng Wang, Min Guan

**Affiliations:** ^1^ Center for Human Tissues and Organs Degeneration Institute of Biomedicine and Biotechnology Shenzhen Institutes of Advanced Technology Chinese Academy of Sciences Shenzhen 518055 Guangdong China; ^2^ High Magnetic Field Laboratory Key Laboratory of High Magnetic Field and Ion Beam Physical Biology Chinese Academy of Sciences Institute of Physical Science and Information Technology Anhui University Hefei 230031 Anhui China; ^3^ Center for Biomedical Materials and Interfaces Institute of Biomedicine and Biotechnology Shenzhen Institutes of Advanced Technology Chinese Academy of Sciences Shenzhen 518055 Guangdong China

**Keywords:** bone apatite, citrate, metabolism, osteogenic differentiation, zinc

## Abstract

Citrate is essential to biomineralization of the bone especially as an integral part of apatite nanocomposite. Citrate precipitate of apatite is hypothesized to be derived from mesenchymal stem/stromal cells (MSCs) upon differentiation into mature osteoblasts. Based on ^13^C‐labeled signals identified by solid‐state multinuclear magnetic resonance analysis, boosted mitochondrial activity and carbon‐source replenishment of tricarboxylic acid cycle intermediates coordinate to feed forward mitochondrial anabolism and deposition of citrate. Moreover, zinc (Zn^2+^) is identified playing dual functions: (i) Zn^2+^ influx is influenced by ZIP1 which is regulated by Runx2 and Osterix to form a zinc‐Runx2/Osterix‐ZIP1 regulation axis promoting osteogenic differentiation; (ii) Zn^2+^ enhances citrate accumulation and deposition in bone apatite. Furthermore, age‐related bone loss is associated with Zn^2+^ and citrate homeostasis; whereas, restoration of Zn^2+^ uptake alleviates age‐associated declining osteogenic capacity and amount of citrate deposition. Together, these results indicate that citrate is not only a key metabolic intermediate meeting the emerging energy demand of differentiating MSCs but also participates in extracellular matrix mineralization, providing mechanistic insight into Zn^2+^ homeostasis and bone formation.

## 1 Introduction

Differentiation and mineralization during osteoblastogenesis of mesenchymal stem/stromal cells (MSCs) are requisites to building bone.^1^ Osteoblastogenesis precedes propagation of mineral onto an extracellular matrix (ECM) consisted of collagen fibrils, osteocalcin, and osteopontin (OPN) secreted by the differentiating MSCs.^2^ Nanocrystal nucleation of apatite subsequently forms on such mineralized ECM. By means of advanced solid‐state NMR spectroscopy and distance measurements, citrate is found strongly bound to the apatite nanocrystals in bone and accounts for about 5 wt% of bone organic fraction.^3^ This vast amount of citrate with its carboxylate groups, provides a huge capacity for calcium binding on apatite; thereby, stabilizing the size of nanocrystal and forming the biomineralizated network essential for bone stability, strength, and resistance to fracture.^3^, ^4^ However, the source of citrate in bone is not clearly known; in particular, how intracellular citrate metabolism and deposition is governed during apatite formation is not well defined.

As a key intermediate in mitochondrial tricarboxylic acid (TCA) cycle, citrate is produced from acetyl‐coA and oxaloacetate by citrate synthase (CS), converted to isocitrate by mitochondrial aconitase (ACO2), and then oxidized to α‐ketoglutarate by isocitrate dehydrogenase 2 (IDH2). Citrate homeostasis is critically important for energy production and survival; therefore, citrate homeostasis must be tightly controlled. Intriguingly, the activity of citrate metabolizing enzyme ACO2 is inhibited by a high level of zinc (Zn^2+^), leading to citrate accumulation and eventual secretion into the ECM, highlighting a connection between Zn^2+^ and citrate homeostasis.^5^ The majority of Zn^2+^ in the body is stored in the skeleton and released during bone resorption.^6^ Furthermore, Zn^2+^ acts as an activator or coactivator of a variety of proteins involved in osteogenesis including the earliest determinant of osteoblast differentiation runt‐related transcription factor 2 (Runx2) and its downstream target gene Osterix, a zinc finger motif containing transcription factor.^7^ Additionally, incorporation of zinc into biomaterials has been observed to enhance bone formation and mineralization.^8^ These observations indicate that Zn^2+^ homeostasis might contribute to citrate accumulation and deposition in bone apatite.

MSCs‐differentiated osteoblasts have a characteristic secretory function with regard to calcium phosphate‐containing matrix vesicle secretion for bone apatite formation.^9^ Here, we ask if citrate production and deposition is associated with this MSC‐mediated biomineralization process and how citrate metabolism is coordinately regulated by Zn^2+^ during bone remodeling. By ^13^C‐isotope‐labeled glucose ((U‐^13^C_6_)‐Glucose) tracing experiments, we found that glucose‐derived mitochondrial citrate is deposited in apatite at the late stage of osteoblast differentiation of MSCs. Notably, orchestrated regulation of Runx2/Osterix and Zn^2+^ uptake promoted citrate accumulation and apatite formation which is found to be in decline upon aging leading to degenerated bone formation.

## 2 Results

### 2.1 Citrate Deposition during Osteoblast Differentiation of hMSCs for Apatite Formation

**Figure 1:**
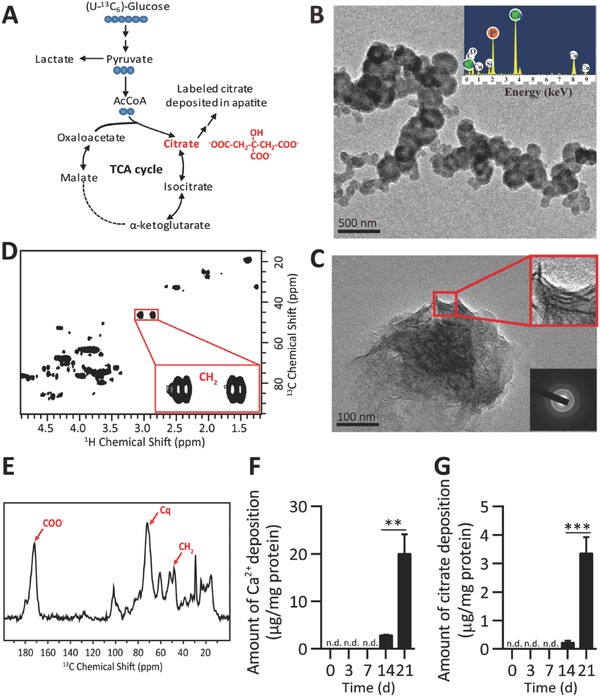
Determination of (U‐^13^C_6_) glucose‐derived citrate deposition during osteogenic differentiation and mineralization of hMSCs. A) Graphical depiction of glucose metabolized into the TCA cycle. Citrate was ^13^C labeled as a metabolite intermediate in MSCs osteogenic cultures supplied with (U‐^13^C_6_)‐Glucose. B) Bright‐field TEM images and the EDX analysis (inset) revealed the formation of calcium phosphate minerals within the differentiated hMSCs nodules. (Scale bar, 500 nm.) C) Mineralization on collagen fibrils (upper inset) in the extracellular space of a mineralized nodule formed from differentiated hMSCs. (Scale bar, 100 nm.) D) Liquid‐phase ^1^H‐^13^C HSQC spectrum of acid extracts from precipitations of mineralized MSCs with ^13^C‐labled glucose supplement (highlights are resonances of the citrate CH_2_ groups). E) Solid‐state ^13^C cross‐polarization NMR spectrum of the mineralized MSCs supplied with (U‐^13^C_6_)‐Glucose confirm the presence of ^13^C‐labeled citrate in apatite of mineralized MSCs. F–G) The amount of calcium (Ca^2+^) F) and G) citrate deposition of differentiated hMSCs at indicated time points. *n* = 4. ***P < 0.001; n.d., not detected.

Bone formation is an energy‐consuming metabolic process in which there are major events of bone matrix synthesis by osteoblasts using glucose as the main nutrient.^10^ We first investigated the relationship between citrate deposition and mineralization by using a well utilized system in which human MSCs (hMSCs) are induced to undergo osteogenic differentiation and tracing with (U‐^13^C_6_)‐glucose (**Figure** **1**A, FigureS1A, Supporting Information). Shown in Figure 1B is the bright‐field transmission electron microscopy (TEM) image of osteogenic hMSC nodules at day 21. X‐ray spectroscopy (EDX) analysis of the material within nodules determined the presence of calcium, phosphorous, indicating that the ECM became mineralized (Figure 1B). Extensive mineralization was also observed in the collagen fibrils associated with the nodules (Figure 1C, upper inset), and the electron diffraction displayed a clear, textured crystalline diffraction pattern (Figure 1C, lower inset). Importantly, we clearly demonstrated that the source of citrate deposited within the mineralized ECM is derived from glucose metabolized in differentiated MSCs through detecting the presence of citrate in acid extracts of mineral precipitates by liquid‐phase ^1^H‐^13^C HSQC spectrum (Figure 1D) and the citrate peaks in mineral precipitates by in situ solid‐state ^13^C cross‐polarization (CP) NMR (Figure 1E). Meanwhile, the mRNA expression levels of two master transcriptional regulators Runx2 and Osterix were significantly upregulated upon differentiation as well as essential constituents of bone ECM collagen type I alpha 1 (Col1A1), osteopontin (OPN), and fibronectin (Figure S1B, Supporting Information). We found that calcium quantitatively deposited in ECM at later stages of differentiation concurrently with citrate (Figure 1F,G) which was recaptured in murine osteoblastic progenitors MC3T3‐E1 cells (Figure S1C,D, Supporting Information). These data suggest that mitochondrial citrate derived from osteoblastogenic differentiated MSCs participates in forming nanocrystals, such as biological apatite.

### 2.2 Citrate Metabolism during Differentiation and Mineralization of hMSCs

**Figure 2:**
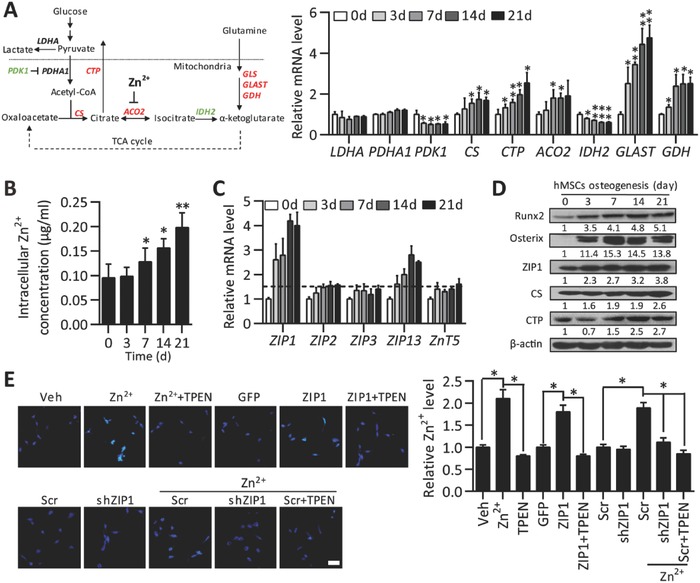
Citrate metabolism and ZIP1‐mediated Zn^2+^ influx during osteogenic differentiation. A) Schematic diagram of metabolic processes involved in citrate accumulation (left panel, downregulated genes marked in green; upregulated genes marked in red). qPCR analysis of mRNA levels of indicted metabolic genes (right panel) (*n* = 4). B) Intracellular Zn^2+^ concentrations in osteogenic differentiating hMSCs were measured by ICP at indicated time points. *n* = 4. C) mRNA levels of Zinc transporters were determined by qPCR analysis. *n* = 4. *P < 0.05; **P < 0.01. compared to control (0 d). D) Protein expression levels were analyzed by western blots analysis during osteogenic induction of hMSCs. E) Cellular levels of Zn^2+^ were imaged (left panel) and quantified (right panel) in MC3T3‐E1 cells treated for 2 d with ZnCl_2_ (Zn^2+^) (20 × 10^−6^ m), TPEN (4 × 10^−6^ m) or control (vehicle); infected with Leni‐Zip1, Lenti‐shZip1, or control (Lenti‐GFP or Lenti‐Scr respectively), with or without Zn^2+^ or TPEN. (Scale bar, 100 µm). *n* = 4. *P < 0.05.

To further understand the metabolic changes in differentiating hMSCs contributing to citrate deposition, we profiled the expression of key enzymes involved in mitochondrial citrate synthesis and catabolism (**Figure** **2**A, left panel). Compared to relatively stable levels of LDHA and PDHA1, we found that PDK1 expression level was significantly decreased upon osteogenic induction (Figure 2A, right panel). Since PDK1 is a negative regulator of pyruvate dehydrogenase (PDH), our results imply an elevation in acetyl‐CoA production. Additionally, we determined that expression levels of CS and mitochondrial citrate transport carrier (CTP) were gradually increased upon osteogenic induction (Figure 2A). Although the expression level of ACO2 was moderately enhanced, the expression level of IDH2 was significantly reduced (Figure 2A). Interestingly, we found the expression levels of glutamine anaplerosis‐associated genes solute carrier family 1 member 3 (SLC3A1 or GLAST) and glutamate dehydrogenase (GDH) were upregulated upon osteogenesis (Figure 2A) in addition to glutaminase (GLS) as we previously reported.^11^ Collectively, these data suggest that glutamine‐dependent anaplerosis is increased favoring production of α‐ketoglutarate which can then be fed into the TCA cycle to be converted to oxaloacetate. The increased acetyl‐CoA production from pyruvate plus additional amount of oxaloacetate from glutamine‐dependent anaplerosis together with elevated CS expression should greatly favor the production of citrate. Moreover, citrate should be further stabilized via suppressing its subsequent oxidation to α‐ketoglutarate by downregulating IDH2. Finally, enhanced CTP expression should facilitate exporting citrate out of the mitochondria for eventual deposition in the calcified ECM.

### 2.3 A Feed Forward Regulation Loop Between ZIP1‐Mediated Zn^2+^ Influx and Runx2/Osterix Accumulation during Osteogenic Differentiation

As Zn^2+^ acts as a competitive inhibitor of ACO2 contributing to citrate accumulation,[[qv: 5a‐c]] we found that intracellular Zn^2+^ was increased constantly during osteogenesis, as well as the amount of Ca^2+^ (Figure 2B, Figure S1E, Supporting Information). Zn^2+^ homeostasis is primarily regulated by membrane Zn^2+^ transporters of the Slc30a family (ZNT) of exporters and Slc39a family (ZIP) of importers.^12^ We then examined the expression levels of several metal zinc‐ion transporters implicated in bone formation.[[qv: 12b,c,13]] We found that ZIP2, ZIP3, and ZnT5 mRNA levels were relatively unchanged (<1.5 fold) (Figure 2C). However, mRNA level of Zip13 which is responsible for intracellular Zn^2+^ distribution was enhanced after day 7 upon osteogenic induction (Figure 2C). Of note, mRNA and protein levels of ZIP1 which is localized to the cell membrane and uptakes Zn^2+^ were markedly induced after day 3 upon osteogenic induction (Figure 2C,D; Figure S2A,B, Supporting Information), making it the most dominantly expressed zinc influx mediator among these zinc‐ion transporters in osteogenic differentiated hMSCs. This prompted us to investigate the transport efficiency of Zn^2+^ by ZIP1 and the regulation of ZIP1 at the cellular microenvionmental context of osteoblasts. First, we examined the uptake of Zn^2+^ supplementation in murine osteoblastic MC3T3‐E1. Zn^2+^ influx was significantly increased with addition of ZnCl_2_ and abolished by a cell‐permeable chelator *N*,*N*,*N*′,*N*′‐tetrakis (2‐pyridylmethyl) ethylenediamine (TPEN) (Figure 2E) or a cell‐impermeable metal ion chelator calcium‐saturated ethylenediaminetetraacetic acid (CaEDTA) (data not shown). We then characterized the regulation of Zn^2+^ influx by modulation of ZIP1 expression using a lentiviral system. Overexpression of ZIP1 in osteoblasts caused significant Zn^2+^ influx which was blocked by TPEN. On the contrary, downregulation of ZIP1 with lentivius‐shRNA (shZIP1) significantly blocked the ZnCl_2_‐induced Zn^2+^ influx as efficiently as TPEN treatment (Figure 2E), demonstrating the essential role of ZIP1‐mediated Zn^2+^ influx in osteoblastic cells.

**Figure 3:**
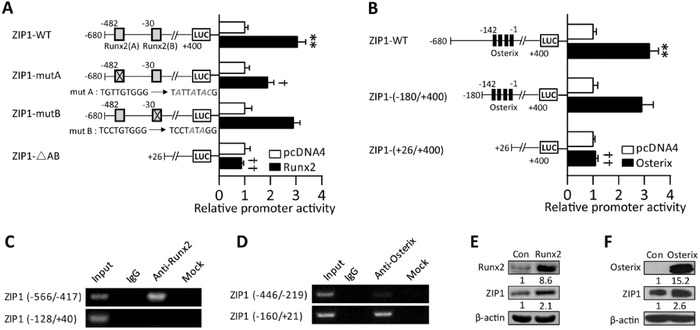
Transcriptional regulation of ZIP1 by Runx2 and Osterix. A,B) Effect of Runx2 A) or Osterix B) on wild‐type human ZIP1 promoter, deletion, and mutation constructs activity by luciferase reporter assay in MC3T3‐E1 cells. *n* = 6. **P < 0.01 compared with control pGL3‐basic; ^†^ *P* < 0.05 and ^††^ *P* < 0.01 compared to ZIP1‐WT. C,D) Chromatin immunoprecipitation analysis of Runx2 C) and Osterix D) response elements on ZIP1 promoter in osteogenic differentiated hMSCs. No antibody was used for mock, whereas an IgG antibody was used as a negative control. E,F) Western blots analysis of the effect of overexpressing E) Runx2 and F) Osterix on ZIP1 protein in MC3T3‐E1.

Zn^2+^ influx causes activation of Zn^2+^‐sensing, metal‐dependent transcription factors. In particular, Zn^2+^ has been shown to stimulate the expression of osteogenic master regulators Runx2 and Osterix.^7^ We confirmed that supplementing Zn^2+^ enhanced mRNA and protein levels of Runx2 and Osterix which was blunted by shZIP1 or TPEN (Figure S2C, Supporting Information). Additionally, ZIP1 expression pattern was similar to Runx2 and Osterix (Figure 2C,D; Figure S1B, Supporting Information). This prompted us to investigate whether Runx2 or Osterix directly regulates ZIP1 expression. Bioinformatics analysis of human ZIP1 promoter region spanning −680 to +400 base pair (bp) upstream of transcriptional start site revealed potential Runx2‐ and Osterix‐binding sites. Referred to as sites A and B, two putative binding sites of Runx2 are located at positions −482 to −469 bp and −30 to −22 bp, respectively. There are four predicted GC/GT boxes recognized by Osterix clustered at position −142 to −1 bp. By using luciferase reporters assay in MC3T3‐E1 cells, we confirmed that overexpression of either Runx2 or Osterix enhanced the wild‐type human ZIP1 promoter activity (**Figure** **3**A,B). The ZIP1 promoter–reporter construct with A site mutated or AB deleted, but not the construct with B site mutated, had significant decreased promoter's activity in Runx2‐overexpressing cells (Figure 3A). Additionally, the construct containing four predicted GC/GT boxes showed similar activity as pGL3‐hZIP1‐WT, while deleted construct (+26/+400) had completely lost its activity in Osterix‐overexpressing cells (Figure 3B). To further confirm whether Runx2 or Osterix can physically bind to response elements in the promoter region of ZIP1 in differentiating hMSCs, chromatin immunoprecipitation assay was performed. Polymerase chain reaction (PCR) amplification showed that a fragment containing site A was immunoprecipitated with Runx2 antibody; a fragment from −160 to +21 bp containing four GC/GT boxes was immunoprecipitated with Osterix antibody (Figure 3C,D). Moreover, overexpression of both Runx2 and Osterix further enhanced the protein expression level of ZIP1 (Figure 3E,F). Collectively, our analysis indicates that Runx2 and Osterix transcriptionally control ZIP1 expression through bona fide Runx2‐ and Osterix‐binding sites in the ZIP1 promoter.

### 2.4 The Zn^2+^ Influx Mediator ZIP1 Coordinated with BMP2/Runx2/Osterix to Promote Osteogenesis and Citrate Deposition

**Figure 4:**
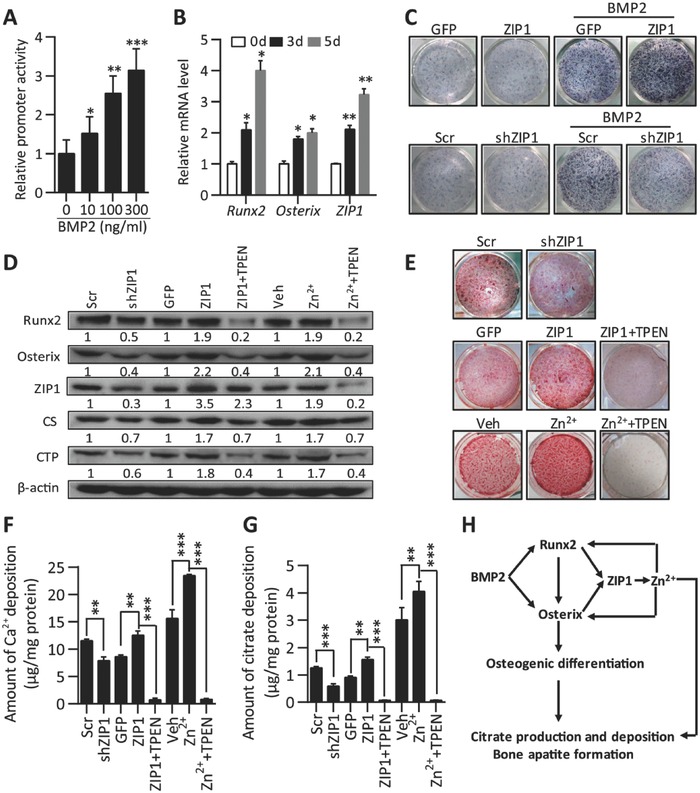
A positive feed‐forward zinc‐Runx2/Osterix‐ZIP1 regulation loop promotes osteogenic differentiation and citrate deposition. A) The effect of BMP2 on ZIP1 promoter activity at indicated doses. *n* = 6. B) mRNA levels of Runx2, Osterix, and ZIP1 were measured by qPCR at 0, 3, and 5 d in hMSCs treated with BMP2 (100 ng mL^−1^). *n* = 4. C) ALP staining analysis for the differentiation potentials of hMSCs transducted by Lenti‐ZIP1, shZIP1 or control lenti‐GFP, shRNA at a MOI of 30; with or without BMP2 (100 ng mL^−1^) treatment for 3 d. D) Western blot analysis of protein expression levels on day 7 of osteogenic induction in hMSCs treated with Zn^2+^ (20 × 10^−6^ m) and vehicle; or infected with Leni‐Zip1, Lenti‐shZip1, and control (Lenti‐GFP or Lenti‐Scr respectively); with or without treatment of TPEN (4 × 10^−6^ m). E) Alizarin red S staining analysis of mineralized hMSCs treated as in (D) at day 21. F,G) The amount of Ca^2+^ F) and citrate G) deposited in differentiated hMSCs at day 21 as in (E). *n* = 4. *P < 0.05, **P < 0.01, and ***P < 0.001 versus control. H) A schematic diagram illustrating the feed‐forward mechanism for how zinc‐Runx2/Osterix‐ZIP1 regulation loop promotes osteogenic differentiation and citrate deposition for bone apatite formation.

BMP2, a member of the transforming growth factor‐β superfamily, has a unique role in MSC differentiation by controlling the transition from progenitors to Runx2^+^ Osterix^+^ cells.[[qv: 7b,14]] We found BMP2 upregulated ZIP1 promoter activity dose dependently (**Figure** **4**A) acting synergistically together with Runx2 or Osterix (Figure S3A, Supporting Information). Consistently, mRNA expression level of ZIP1 was also enhanced after treating hMSC in growth medium supplemented with BMP2 for indicated days, as well as Runx2 and Osterix (Figure 4B). To verify ZIP1 is required for BMP2 signaling and affects osteogenic differentiation, we assayed for alkaline phosphatase (ALP) which is a marker for osteogenic differentiation. We demonstrated that knockdown of ZIP1 significantly attenuated the osteogenic differentiation; whereas, overexpression of ZIP1 promote differentiation with treatment of BMP2 (Figure 4C, FigureS3B, Supporting Information). We next explored the role of ZIP1‐mediated Zn^2+^ influx in MSC mineralization and citrate deposition. First, we determined that Runx2 and Osterix were downregulated with knockdown of ZIP1 expression during hMSC osteogenesis. In contrast, overexpression of ZIP1 or Zn^2+^ supplement has the opposite effect which was blocked by TPEN (Figure 4D). By determination of Alizarin red S staining and calcium deposition, we further confirmed that knockdown of ZIP1 decreased osteoblast mineralization (Figure 4E,F). Notably, overexpression of ZIP1 or Zn^2+^ supplementation promoted mineralization, an effect that was abolished by TPEN (Figure 4E,F). However, elevated Zn^2+^ levels had no effect on adipogenic differentiation of hMSC, highlighting its specific function on fate determination of MSC (Figure S3C, Supporting Information). Since Zn^2+^ has dual functions of inhibiting ACO2 activity and promoting osteogenic differentiation, we reasoned it might accelerate citrate production and deposition to form calcified ECM during MSC mineralization. Indeed, both of ZIP1‐mediated Zn^2+^ influx and Zn^2+^ supplementation significantly increased citrate deposition that was abolished by TPEN; whereas, downregulation of ZIP1 deteriorated citrate deposition of mineralized ECM (Figure 4G). These results collectively suggest that the Zn^2+^ influx mediator ZIP1 coordinates with BMP2/Runx2/Osterix to promote osteogenic differentiation, meanwhile Zn^2+^ influx enhances citrate deposition and bone apatite formation (Figure 4H).

### 2.5 Age‐Related Bone Loss is Associated with Zn^2+^ and Citrate Homeostasis

**Figure 5:**
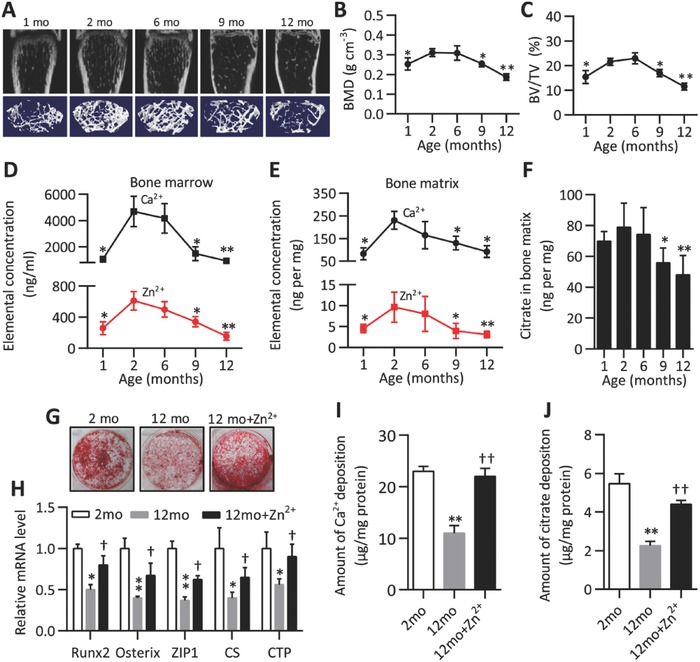
An increase in Zn^2+^ influx during osteogenesis attenuates citrate deposition loss with aging. A) Representative microCT images of trabecular microarchitecture of secondary spongiosa in femora from male mice at 1, 2, 6, 9, and 12 months of age. B,C) Quantification of B) trabecular bone density and C) bone volume. *n* = 6. (D,E) Ca^2+^ and Zn^2+^ concentrations in D) bone marrow and E) matrix extractions were measured by ICP. *n* = 6. F) The concentrations of citrate in bone matrix extractions of indicated ages of mice. *n* = 6. G) Alizarin red S staining analysis of differentiated mMSCs derived from 2‐ and 12‐mo mice with or without Zn^2+^ (20 × 10^−6^ m) treatment for 21 d. H) qPCR analysis of mRNA levels of indicted genes on day 7 of osteogenic induction in mMSCs as in (G). I,J) The amount of I) Ca^2+^ and J) citrate deposited in differentiated mMSCs as in (G). *n* = 4. *P < 0.05 and **P < 0.01 compared to 2 months of age; ^†^ *P* < 0.05, and ^††^ *P* < 0.01. compared to 12 months of age.

Differentiated and mineralized MSC at bone‐resorptive sites contribute to new bone formation during bone remodeling.^15^ Of note, in human, Zn^2+^ and Ca^2+^ concentrations were also correlated with osteopenia due to estrogen deficiency or aging.^16^ We next asked if the amount of citrate was correlated with the changes in bone mass. We first assessed bone volume and bone mass of wild‐type male mice at ages 1, 2, 6, 9, and 12 months. Both parameters increased steadily from 1 to 2 months after birth; then, declined continuously from 6 months of age onward and reached a marked decrease at 12 months of age (**Figure** **5**A–C). Consistently, changes in the concentrations of Zn^2+^ and Ca^2+^ in the bone marrow and matrix of the mice were well correlated with the changes in bone mass described above (Figure 5D,E). Notably, citrate concentration in the bone matrix also showed similar trend with Ca^2+^ and Zn^2+^ (Figure 5F). This correlation suggests a crucial role for bone matrix citrate in the maintenance of bone mass, as well as Zn^2+^ and Ca^2+^. These data further support the notion that production and deposition of citrate was closely associated with Zn^2+^ homeostasis during bone formation.

### 2.6 An Increase in ZIP1/Zn^2+^ Attenuated Age‐Associated Loss of Matrix Citrate Deposition

To determine whether aging affects the uptake of Zn^2+^ of MSC which in turn influences osteogenesis and citrate metabolism, we isolated bone marrow MSC from mice at 2 and 12 months of age for an ex vivo analysis. We found MSC isolated from aged (12 months of age) mice exerted an apparent decrease in osteogenic mineralization and citrate deposition (Figure 5G,I); reduction in mRNA expression levels of ZIP1, CS, and CTP as well as Runx2 and Osterix (Figure 5H). These results indicate that ZIP1 mediated Zn^2+^ influx is correlated with osteogenic capacity and citrate deposition of MSC upon aging. Thus, we further confirmed that the anabolic role of ZIP1/Zn^2+^ in improving osteogenic capacity of MSC upon aging. We found that rescued ZIP1 expression or Zn^2+^ supplement highly improved osteogenic capacity and citrate deposition as well as enhancing expression levels of indicated genes (Figure 5G–J, Figure S4A–F, Supporting Information). Since long‐term culturing of human MSCs and in vitro reduces their multipotency promoting senescence,^17^ we showed that late‐passage hMSCs showed similar osteogenesis and citrate metabolism patterns to MSC from aged mice, recapitulating features of Zn^2+^ influx of MSCs upon aging (Figure S5A–F, Supporting Information). Taken together, these observations indicate that Zn^2+^ levels are positively correlated with age‐related MSCs differentiation and mineralization, consistent with our findings that ZIP1‐mediated Zn^2+^ influx and citrate deposition favor bone apatite formation.

## 3 Discussion

Although citrate has been found universally presented in vertebrate bone and accounts for ≈80% of all citrate in the body for several decades,^18^ only lately has it been demonstrated as a strongly bound, integral part of the nanocomposite in bone.^3^ Here, we reported that citrate of bone apatite was produced by mineralized MSCs by tracing with stable isotopically labeled carbon (Figure 1). Metabolomics analysis of differentiated MSC proved that glycolytic induction of lactate production is lowered; whereas, the amount of mitochondrial TCA intermediates such as citrate derived from oxidative metabolism are increased.^19^ A novel model for bone apatite formation proposed that calcium and phosphorus‐containing mineral aggregates in osteoblast mitochondria, transports via vesicles to the ECM and then converts to more crystalline.^9^ Interestingly, we determined that accumulation of citrate precipitation was closely associated with calcium deposition and ZIP1‐mediated Zn^2+^ influx during osteogenesis (Figures 1, 4, and 5). These evidences imply that the transport of calcium and possibly citrate take place in mitochondrial and intracellular compartments during osteogenesis, providing a missing link in deciphering the process of normal bone apatite formation.

Mitochondrial regulation plays crucial roles in differentiation of pluripotent stem cells.^20^ Growing evidence shows that during MSC differentiation, mitochondria undergo significant dynamics and bioenergetics changes.^21^ For instance, we previously observed that mitochondrial biogenesis‐associated genes, an essential regulator ERRα and its coactivator PGC‐1α, induce glutamine anaplerosis and ramp up α‐ketoglutarate production feeding into the TCA cycle during osteogenesis.^11^ In this study, we further confirmed that extracellular citrate deposition arises from mitochondrial TCA metabolism (Figure 2). To account for the increased production of citrate, we found that genes involved in citrate anabolism and transportation CS and CTP were induced; whereas, IDH2 was suppressed, suggesting a coordinated regulation to promote citrate production while reducing citrate catabolism. Furthermore, glutaminolysis‐associated genes GLS, GLAST and GDH were significantly enhanced, implying a surge of α‐ketoglutarate for feeding forward the TCA cycle for more citrate production. Although we use (U‐^13^C_6_)‐glucose as the only labeled tracer in this study, we propose that other carbon sources, such as amino acids or fatty acids can enter into the TCA cycle and contribute to citrate production. Collectively, our data suggest that boosted mitochondrial activity and carbon‐source replenishment of TCA intermediates are coordinated to feed forward mitochondrial anabolism of citrate.

In addition to apparently activated oxidative metabolism during osteogenic differentiation of MSCs, an upregulation of zinc influx likely suppresses ACO2 activity reducing citrate catabolism[[qv: 5c,22]] and further promote citrate accumulation and deposition (Figures 2, 3, 4). Clinically, age‐related osteoporotic patients have lower levels of skeletal zinc.[[qv: 6b,16b]] Zn^2+^ deficiency causes low bone mass.^23^ Supplementation of nutritional Zn^2+^ has an anabolic therapeutic effect on bone loss.[[qv: 6a,24]] Similar to Ca^2+^ and other bone formation stimulating factors,[[qv: 6b,25]] Zn^2+^ is also released from bone resorptive sites during bone remodeling,^26^ generating a localized high Zn^2+^ concentration environment. Here, we observed that an uncoupling of bone remodeling with marked changes of citrate production and deposition, as well as Zn^2+^ and Ca^2+^ (Figure 5). We determined that intracellular zinc content gradually accumulated and further identified that ZIP1 was one dominant zinc transporter required for zinc uptake during osteogenesis of MSCs (Figure 2). Consistent with others report,[[qv: 7b]] we showed that Runx2 and Osterix, two key transcription factors for osteogenic differentiation, are both induced by Zn^2+^ influx. Of note, Runx2 and Osterix transcriptionally regulated ZIP1 expression which further leads to induction of Zn^2+^ influx contributing to a positive feed‐forward zinc‐Runx2/Osterix‐ZIP1 regulation loop during osteogenic differentiation (Figures 3 and 4). Importantly, this regulatory loop is perturbed with aging, contributing to the declined osteogenic capacity of MSC and degenerated bone formation. In contrast, addition of Zn^2+^ or overexpression of ZIP1 caused enhancement of aged MSC differentiation and restoration of citrate and Ca^2+^ deposition in mineralized MSCs (Figure 5). Further studies will be required to define intracellular metabolic changes of citrate and its deposition during uncoupling bone remodeling under conditions of other bone diseases, such as estrogen deficiency‐related osteopenia. It will be of great interest to explore whether Zn‐incorporated biomaterials simulate citrate‐containing bone apatite formation which might contribute to osteointegration process of cells‐biomaterials interface and bone regeneration.[[qv: 8b,c]]

## 4 Conclusion

**Figure 6:**
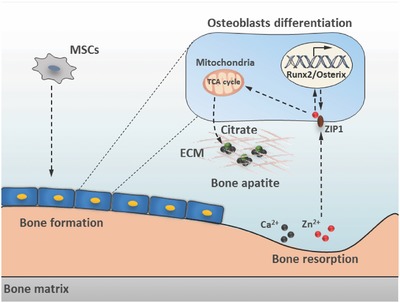
Diagram of Zn^2+^ induced osteoblast differentiation of MSCs and citrate deposition during bone remodeling. During bone remodeling, mineral elements/ions released from the bone matrix comprising the osteogenic microenvironment for differentiation of MSCs. We proposed that zinc (Zn^2+^) has dual functions: (i) Zn^2+^ influx is influenced by ZIP1 which is transcriptionally mediated by Runx2 and Osterix to form a zinc‐Runx2/Osterix‐ZIP1 regulation axis promoting osteoblast differentiation at an early differentiation stage; (ii) Zn^2+^ enhances citrate accumulation and deposition in ECM at mineralization stage. This process is more likely to be associated with calcium (Ca^2+^) deposition for bone apatite formation.

Our results demonstrate that zinc‐Runx2/Osterix‐ZIP1 regulation axis promotes osteoblast differentiation and apatite formation; uncover mitochondrial citrate metabolism and its relationship with zinc homeostasis during bone remodeling (**Figure** **6**). These findings highlights that mitochondrial and metabolic changes not only meet higher amounts of energy demand during osteogenic differentition of MSC, but also provide metabolic intermediates directly participating in bone apatite formation. The imbalance of zinc and citrate homeostasis upon aging lead to deleterious bone formation which may have important implications for understanding and treating pathological bone loss.

## 5 Experimental Section

*Mice*: Male C57BL/6 mice were purchased from Vital River Laboratory Animal Technology Co. Ltd. (Beijing, China). The experimental protocols were approved by the Institutional Animal Care and Use Committee of Shenzhen Institutes of Advanced Technology, Chinese Academy of Sciences.

*MSCs Isolation and Culture*: For mouse primary MSCs isolation, bone marrow cells were flushed out from femora of mice_._ After 24 h incubation, nonadherent cells were removed and adherent cells were replenished with complete growth medium composed of α‐MEM, 10% FBS (Corning), and 1% penicillin/streptomycin (pen/strep, Hyclone). Passages 1 and 2 were used in the following experiments. Human MSCs (Cyagen) were cultured with growth medium as previously described.^11^

*Osteogenic Differentiation*: Osteoblasts were differentiated from mouse MSCs, human MSCs, or MC3T3‐E1 as described.^1^, ^11^ Briefly, cells were incubated in osteogenic induction medium comprised of α‐MEM, 10% FBS, 10 × 10^−3^ m β‐glycerophosphate, 50 µg mL^−1^ ascorbic acid, and 100 × 10^−9^ m dexamethasone (Sigma‐Aldrich). To evaluate osteogenic differentiation, cells were seeded in a 24‐well plate with osteogenic induction medium for 7 d or in growth medium supplemented with BMP2 (100 ng mL^−1^) for 3 d and then assayed for alkaline phosphatase, an early marker of osteogenesis. For ALP staining, cells were fixed and stained with 5‐bromo‐4‐chloro‐3‐indolyl phosphate/nitroblue tetrazolium (Beyotime). For ALP activity, cell were lysed and incubated with p‐nitrophenylphosphate substrate and the absorbance at 405 nm was measured. For Alizarin red S staining, cells were incubated in osteogenic induction medium for 21 d, and then fixed and stained with 2% Alizarin red S (Sigma‐Aldrich) at pH 4.2 to evaluate cell matrix calcium deposition. Unless otherwise indicated, cells were treated with 20 × 10^−6^ m Zncl_2_ (Aladdin) or 4 × 10^−6^ m cell‐permeable zinc chelator *N*,*N*,*N*′,*N*′‐tetrakis (2‐pyridylmethyl) ethylenediamine (Sigma‐Aldrich). For tracing experiment, after 19 d of osteogenic induction, (U‐^13^C_6_)‐Glucose (Cambridge Isotope Laboratories) was added to the medium (no glucose, Gibco) at the final concentration of 1 g L^−1^ for another 3 d in culturing media.

*Electron Microscopy Analysis*: Mineralized MSCs were collected and resuspended in water and solution (10 µL) was transferred to a carbon‐coated copper grid. Bright‐field TEM, selected area electron diffraction and energy dispersive X‐ray spectroscopy investigations were performed with Philips CM200 Field Emission (FEG) TEM operated at an acceleration voltage of 200 kV.

*NMR Experiments*: All liquid state NMR experiments were performed at 298 K on a Bruker AVANCE III 500 MHz spectrometer equipped with a 1.7 mm CPQCI cryoprobe. ^13^C CP/MAS solid state NMR experiments were performed on Bruker AVANCE III 600 spectrometer at a ^13^C resonance frequency of 150.9 MHz. For more NMR details and sample preparation, please see Supporting Information Methods.

*RNA Extraction and Quantitative RT‐PCR Analysis*: Total RNA was extracted from cultured cells using Trizol reagents (Invitrogen). Synthesis of cDNA was performed using 2 µg of RNA by a Transcriptor First Strand cDNA Synthesis Kit (Thermo) according to the manufacturer's instructions. Relative mRNA expression levels were determined by applying a SYBR Green qPCR kit (Transgen Biotech). Values were normalized to β‐actin.

*Western Blot Analysis*: Total cell lysates were prepared in RIPA lysis buffer (150 × 10^−3^ m NaCl, 1% NP‐40, 50 × 10^−3^ m Tris, 5 × 10^−3^ m NaF, 0.1% sodium dodecyl sulfate (SDS)) and aliquots of 60 µg total protein were separated and blotted onto a polyvinylidene fluoride membrane (Millipore). Membranes were blocked at room temperature in 5% nonfat powdered milk in Tris‐buffered saline followed by an overnight incubation at 4 °C with specific antibodies recognizing β‐actin, CS, CTP (Santa cruz #sc‐8432, #sc‐390693, #sc‐86391), ZIP1 (Millipore #ABC849), Runx2 (Cell Signaling Technology #12556), and Osterix (Abcam #ab22552), respectively. After incubation with appropriate horseradish peroxidase (HRP)‐conjugated secondary antibodies (Santa cruz), blots were developed using an enhanced chemiluminescence (ECL Kit, Millipore) and exposed in ChemiDoc XRS chemiluminescence imaging system (Bio‐Rad).

*Plasmids Construct, Lentivirus Production, Luciferase Reporter Assay and Chromatin Immunoprecipitation*: This analysis has been previously described in detail earlier.^11^, ^27^ See Supporting Information Methods for details.

*Bone Analyses*: Right femora were excised and fixed for µCT analysis (SkyScan). Obtained slices were reconstructed and analyzed as previously described.^11^ Bone matrix and marrow extractions were isolated from left femora. We exposed bone marrow at the growth plate of left femora and placed the samples for centrifugation for 15 min at 5000 rpm to obtain marrow supernatants.^25^ We immersed the resected femora in liquid nitrogen and ground it using a biopulverizer (BioSpec), defatted in a 3:1 mixture of methanol and chloroform, and removed all traces of solvent by vacuum. The degreased bone powder was boiled in hydrochloric acid at 80 °C for 24 h to obtain the bone matrix extraction for further analysis.^3^ The concentrations of citrate in the bone matrix extracts were measured using Citrate Assay Kit (BioAssay Systems).

*Assessment of Zinc and Calcium*: The concentrations of zinc and calcium from intracellular, bone marrow, or matrix extractions were determined by inductively coupled plasma atomic emission spectroscopy (ICP, PerkinElmer). The analytical accuracy for analysis was confirmed using a standard reference material (National Center of Analysis and Testing for Nonferrous Metals and Electronic Materials, China). For intracellular zinc imaging, cells were incubated in 20 × 10^−6^ m Zinquin Ester (Santa cruz) for 30 min. The images were captured using an inverted fluorescence microscope and UV filters (Olympus‐IX71).

*Statistical Analyses*: The group means and standard deviations (SDs) were calculated for all outcome variables. Statistical analyses were analyzed using one‐way ANOVA followed by two‐tailed Student's *t*‐test. A difference was considered to be statistically significant at *P* < 0.05.

## Conflict of Interest

The authors declare no conflict of interest.

## Supporting information

Supplementary
