# Supplementary material for: Runx2/Osterix and Zinc Uptake Synergize to Orchestrate Osteogenic Differentiation and Citrate Containing Bone Apatite Formation
Source: Adv Sci (Weinh). 2018 Jan 28;5(4):1700755. doi: 10.1002/advs.201700755 (PMC5908346; doi:10.1002/advs.201700755)
Supplement: Supplementary file 1 — Supplementary [file ADVS-5-1700755-s001.pdf]

Copyright WILEY-VCH Verlag GmbH & Co. KGaA, 69469 Weinheim, Germany, 2018.

ADVANCED SCIENCE
Open Access

Supporting Information

for *Adv. Sci.*, DOI: 10.1002/advs.201700755

Runx2/Osterix and Zinc Uptake Synergize to Orchestrate Osteogenic Differentiation and Citrate Containing Bone Apatite Formation

Xuekun Fu, Yunyan Li, Tongling Huang, Zhiwu Yu, Kun Ma, Meng Yang, Qingli Liu, Haobo Pan, Huaiyu Wang, Junfeng Wang, and Min Guan**

Copyright WILEY-VCH Verlag GmbH & Co. KGaA, 69469 Weinheim, Germany, 2016.

Supporting Information

Runx2/Osterix and Zinc Uptake Synergize to Orchestrate Osteogenic Differentiation and Citrate containing-Bone Apatite Formation

Xuekun Fu, Yunyan Li, Tongling Huang, Zhiwu Yu, Kun Ma, Meng Yang, Qingli Liu, Haobo Pan, Huaiyu Wang, Junfeng Wang, Min Guan**

NMR experiments: Mineralized MSCs were peeled off with a scratcher in cold water and centrifuged at 4,200g, then resuspended with cold extracting solution, 80% methanol aqueous liquids. The sample was frozen in liquid N_2 and heated to 37 ˚C for three cycles of freeze-thaw treatment before storing at -80 ˚C overnight. After thaw-out and ultrasonication, the mineralized MSCs were centrifuged at 13,000g for 10 mins, and the precipitations were resuspended in extracting solution, followed by two rounds of ultrasonication and centrifugation. The total volume of extracting solution used was 5 mL per plate. The pooled supernatants and precipitations were separated and lyophilized respectively for later NMR experiments. The lyophilized supernatants containing metabolites of TCA cycle were dissolved in 50 μL pH 7.4 PBS (136 mM K_2HPO_4 and NaH_2PO_4, molar ratio of 4:1) D_2O buffer containing 0.05% TSP (m/v, sodium 3-trimethlysilyl [2,2,3,3-$^2H_4$] propionate), where D_2O was used as a field lock and TSP as an internal chemical shift reference. The mineral precipitates can be used directly for solid state NMR. ^{13}C CPMAS NMR spectra (Fig. 1E) were recorded at room temperature using a Bruker AVANCE III 600 MHz spectrometer equipped with a 3.2 mm HCN probe. The ^{13}C RF field was 65 kHz during a 1-ms CP contact period. The ^1H RF field was set to 85 kHz during decoupling and amplitude ramping was used to match the 20 kHz spinning rate. The recycle delay was 2.5 s. To characterize the mineral precipitates by liquid state NMR, the precipitation from 80%

methanol extraction were dissolved in 1 M HCl D_2O solution, and grinded with a micro-tissue-grinder. The supernatants after centrifugation were then studied by liquid state NMR. The NMR spectra of the HCl dissolved mineral precipitates and the methanol-extracted supernatant, were acquired at 298 K on a Bruker AVANCE III 600 MHz spectrometer equipped with a 5 mm CPTCI cryoprobe. High-resolution ^1H-^{13}C HSQC spectra were recorded with 2048 points in t_2 and 256 points in t_1 with a recycle time of 1.5 s. GARP decoupling was applied during t_2. The $^1J_{CH}$ was set to 145 Hz, and the spectra widths were 14 ppm and 165 ppm in ^1H and ^{13}C respectively.

Plasmids construct and dual luciferase reporter assays: Human Runx2 and Osterix were cloned into pcDNA4 vector (Invitrogen) as previously described in refs. 11 using primers for Runx2: forward 5'-CTAGCTAGCGGAGGGACTATGGCATCA-AA-3' and reverse 5'-CCGCTCGAGTTCAATATGGTCGCCAAACAG-3'; Osterix: 5'-CCGGAATTCGTTCCCCCAGCTCTCTCCAT-3' and reverse 5'-CCGCTCGA-GGGCTCAGATCTCCAGCAAGTT -3'. Hman ZIP1 gene promoters were cloned into pGL3 luciferase reporter vector (Promega) by PCR using primers forward 1(WT): 5'-CGGGGTACCTACGCAGATATCCTGGAATGC-3'; forward 2(ΔAB):5'-CGGGGTACCCGCGGAGACCATGTAGTGAG-3'; forward 3(-180/+400): 5'-CGGGGTACCCTCGCTCACTCTCCTCAGGT-3' and reverse: 5'-CCGCTCGAGATGTCTTGGGTAGCTTCGTGG -3'. Mutations were introduced into putative ERRa binding sites by PCR-based sitedirected mutagenesis using QuikChange Site-Directed Mutagenesis Kit (Stratagene). Site-directed mutagenesis primers are mutA: forward 5'-GCCTTGTCTTTTTCCTTATTATACGTGC-TGGTGAGGG-3' and reverse: 5'-CCCTCACCAGCACGTATAATAAGGAAAAA-GACAAGGC-3; mutB: forward 5'-TCGTGGGTACGCGGCTCCTATAGGGG-AGGGGGGA-3' and reverse: 5'- TCCCCCCTCCCCTATAGGAGCCGCGTACCC-ACGA -3'. MC3T3-E1 cells were transiently transfected by Lipofectamine 3000 (Invitrogen) according to the manufacturer's instructions with promoter plasmids (10ng) respectively together with Runx2 or Osterix expression vector (20ng) and an internal control Renilla luciferase (1 ng) for 18 hours. For BMP2 (R&D system)

treatment, cells were added with BMP2 (10, 100 or 300ng/ml). The luciferase activities were measured using Dual-Luciferase Reporter Assay System in a GloMax 96 Microplate Luminometer (Promega). Each value of luciferase assay was normalized against Renilla luciferase value to determine relative activity.

Chromatin immunoprecipitation (ChIP): Human MSCs were cultured in osteogenic induction medium for 7 days and then cross-linked with 1% formaldehyde. ChIP assay was performed using a Chromatin Immunoprecipitation Assay Kit (Millipore) as previously described in refs. 11. Analysis of response elements on ZIP1 promoter was determined by PCR using primers for spanning site (-566/-417): forward 5'-TGCCATAAGTAGTCGAGAAGGG-3' and reverse 5'-GAAAGCATTCCCC-TCTTGGGA-3'; (-128/+40): forward 5'-TCCCGATCTCTGATTGCTCCTA-3' and reverse 5'-CCGGCCTAGAGTCGGGAA-3'; (-160/+21): forward 5'-CTCCTCAGGTCGCCTGCT-3' and reverse 5'-CGGCCTAGAGTCGGGAAT-3'; (-446/-219) : forward 5'-GAAGTTGGTCCCAAGAGGGG-3' and reverse 5'-GCATCTGTGGAGGACTGAGG-3'.

Lentivirus production and infection: To construct pWPXLd-ZIP1 vector, human ZIP1 fragment were PCR amplified using primers forward 5'-CGGGGTACCTACGCAGATATCCTGGAATGC-3' and reverse 5'-CCGCTCGAGGTAGCTCCAGTGACTCTCAGACCT-3'. The ZIP1 shRNA (shZIP) or scramble shRNA (Scr) constructs were constructed by ligation of double-stranded oligonucleotide into the pEN_hH1c vector and then recombined with pDSL-hpUGIP to obtain shZIP1 which targeted both human and mouse ZIP1, and primers are: shZIP1, forward 5' -GATCCCCCCTGACTACCTGGCTGCCATATTCA-AGAGATATGGCAGCCAGGTAGTCAGGTTTTTC- 3' and reverse 5'-TCGAGAA-AAACCTGACTACCTGGCTGCCATATCTCTTGAATATGGCAGCCAGGTAGTC-AGGGGG - 3'; Scr, forward 5'-GATCCCCGCCAATCTTCGTCCTTACTGTTTC-AAGAGAACAGTAAGGACGAAGATTGGCTTTTTC - 3' and reverse 5'-TCGAG-AAAAAGCCAATCTTCGTCCTTACTGTTCTCTTGAAACAGTAAGGACGAAGA

TTGGCGGG-3’. Vector particles were produced in 293T cells involving a three plasmid expression system by transient cotransfection as previously described in refs. 11. For lentiviral infections, cells were cultured in basal growth medium until 70% confluence and replaced with fresh medium containing Lenti-GFP, ZIP1, Scr or shZIP1 at multiplicity of infection (MOI) of 30 in the presence of 100 g/ml of protamine sulfate (Sigma) to be exposed for 16 hours, followed by further analyses.

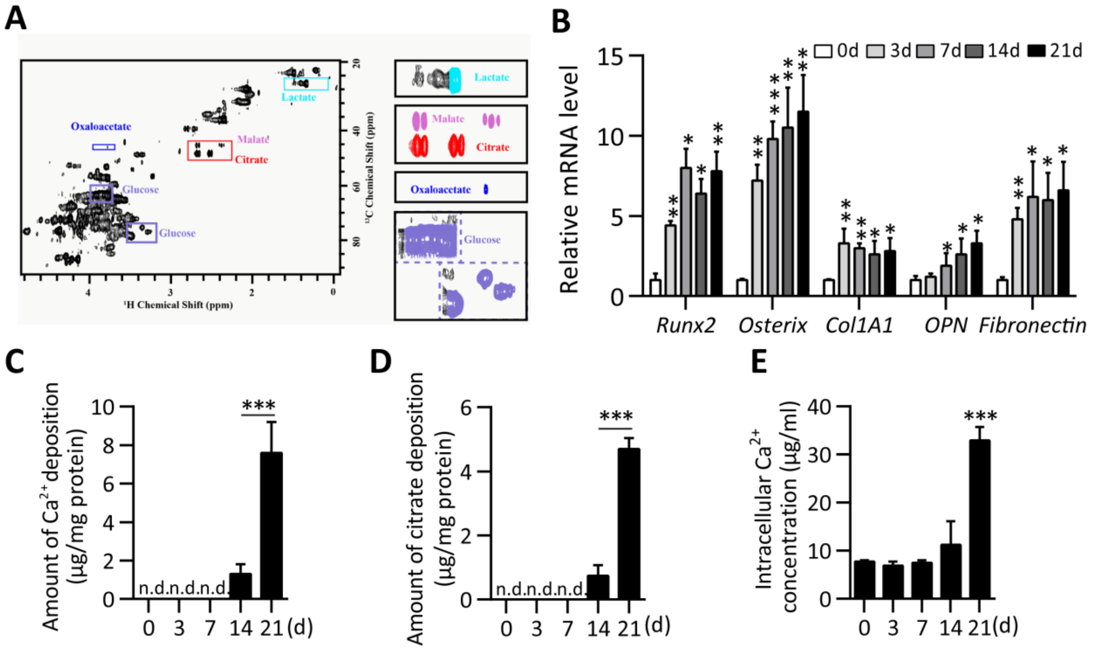

Figure S1. Osteogenic differentiation and mineralization of hMSCs and citrate deposition in differentiated murine osteoblastic progenitors MC3T3-E1.

(A) ^{1}H-^{13}C HSQC spectrum of ^{13}C-labled intracellular metabolic intermediates in differentiated hMSCs (highlighted are resonances from lactate, citrate, oxaloacetate and glucose). (B) qPCR analysis of osteogenic markers and matrix related genes during osteogentic differentiation of hMSCs. (C, D) The amount of Ca^{2+} (C) and citrate (D) deposited in differentiated MC3T3-E1 cells at 0, 3, 7, 14, and 21 day. (E) Intracellular Ca^{2+} concentration in osteogenic hMSCs was detected by ICP. n = 4. *P < 0.05, **P < 0.01, ***P < 0.001; n.d., not detected.

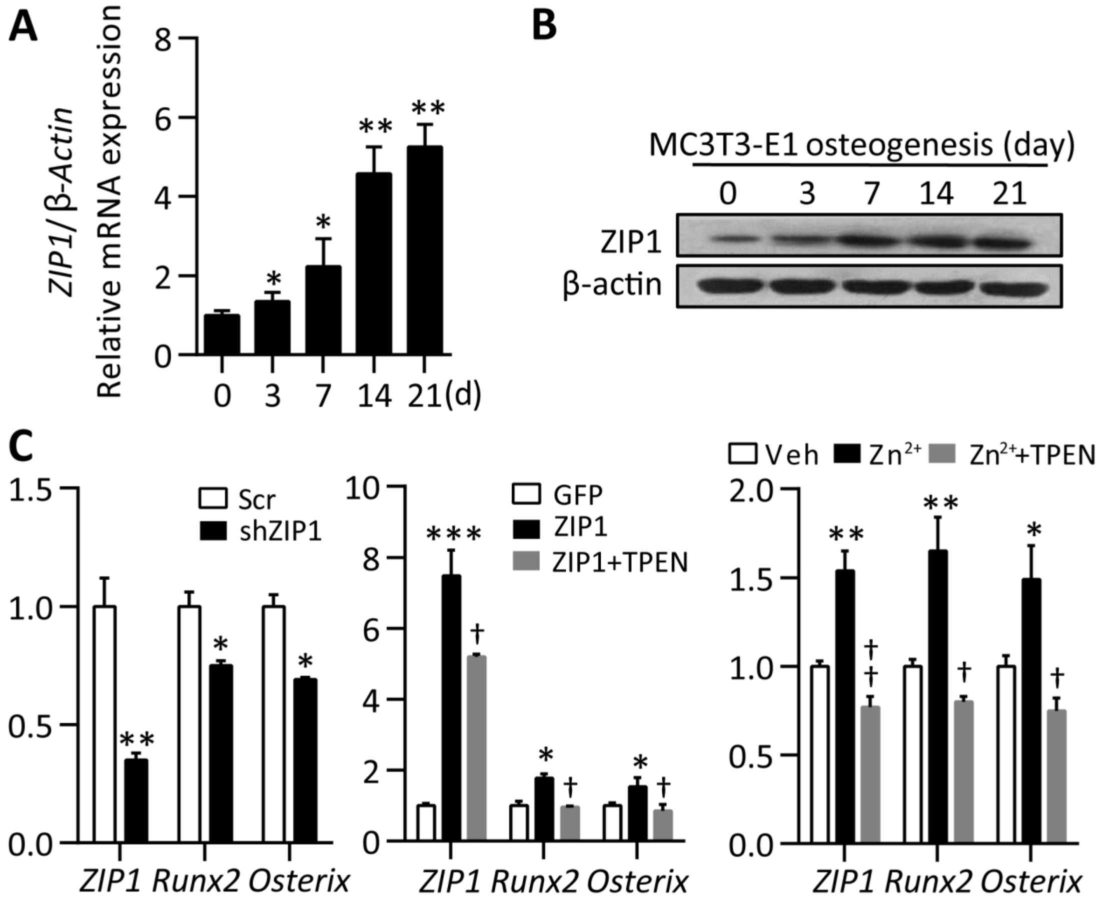

Figure S2. ZIP1/Zn^{2+} promoted osteogenic differentiation of MC3T3-E1. (A, B) qPCR (A) and western blot analysis (B) of ZIP1 expression at 0, 3, 7, 14, and 21 day of osteogenic induction in MC3T3-E1 cells. (C) mRNA levels were measured by qPCR on day 7 of osteogenic induction in MC3T3-E1 cells with indicated treatments. n = 4-6. *P < 0.05, **P < 0.01, ***P < 0.001; †P <0.05 and ††P <0.01 compared to overexpression of Zip1.

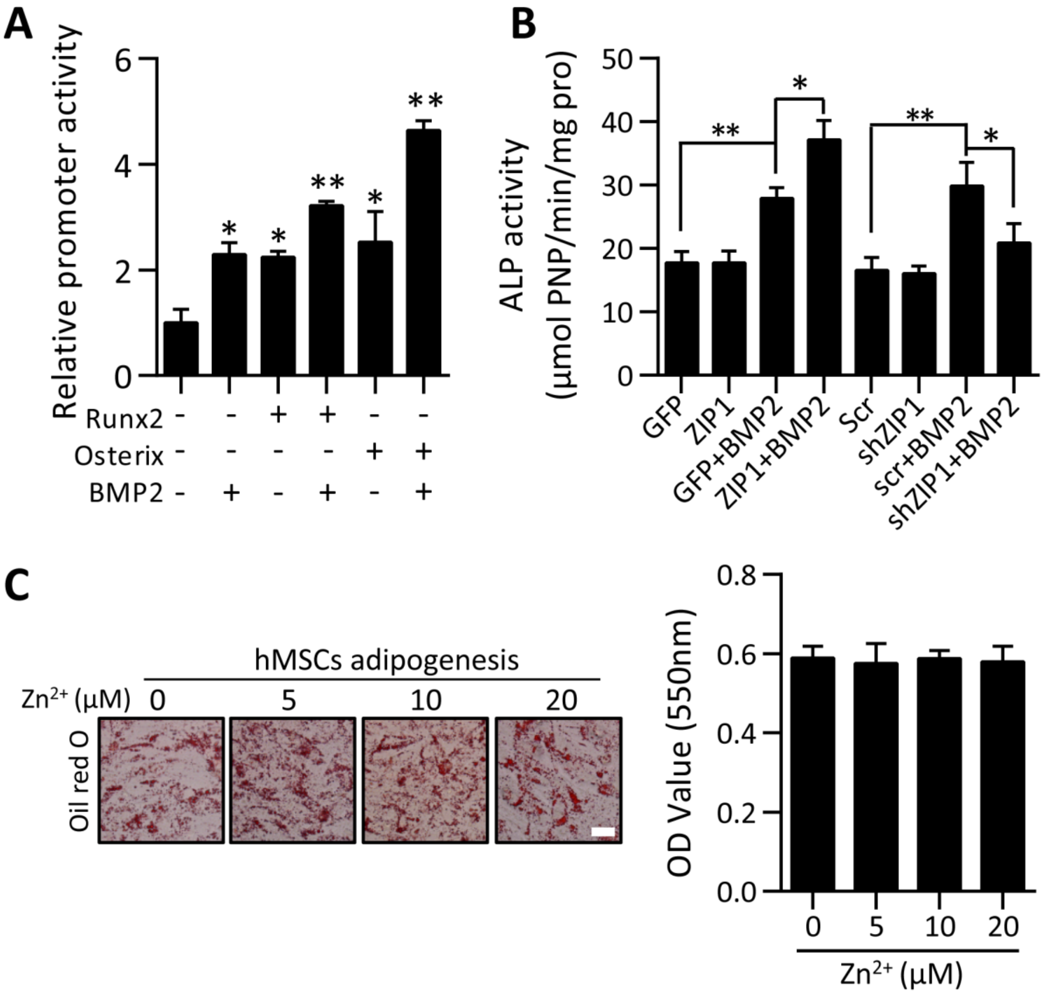

Figure S3. ZIP1/Zn^{2+} coordinated with BMP2 to promote osteogenesis.

(A) MC3T3-E1 cells were cotransfected with pGL3-hZIP1-WT promoter luciferase reporter plasmid with or without overexpression of Runx2, Osterix or BMP2 (100 ng/ml). n = 6. (B) Measurement of ALP activity in hMSCs transfected by Lenti-ZIP1, shZIP1 or control lenti-GFP, shRNA at a MOI of 50; with or without 100 ng/ml BMP2 treatment for 3 days. n = 4. (C) Oil red O staining analysis (left) and quantification (right) of adipogenic differentiation of hMSCs treated with indictated doses of Zn^{2+}. n = 4. *$P < 0.05$, **$P < 0.01$ and ***$P < 0.001$.

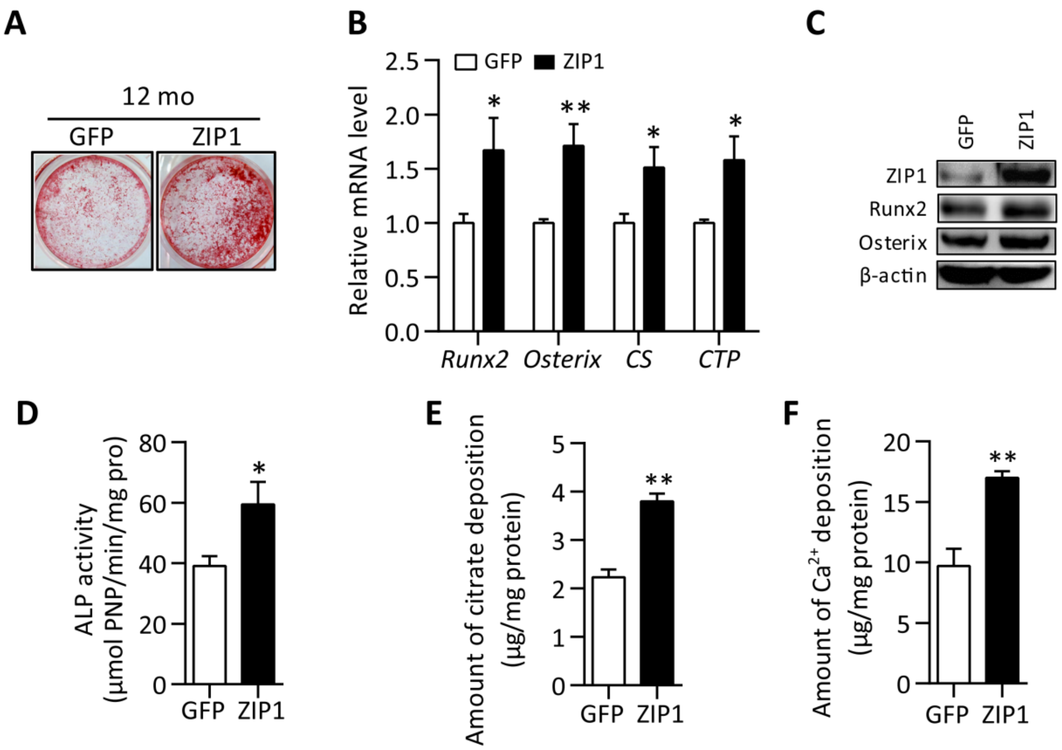

Figure S4. An increase in ZIP1/Zn^{2+} enhanced osteogenic capacity and citrate deposition of MSCs in elderly mice. (A) Alizarin red S staining analysis of differentiated mMSCs derived from 12-month old mice transfected with lenti-ZIP1 or control lenti-GFP (MOI 30). (B, C) qPCR and western blot analysis of the expression levels of indicated genes (B) and proteins (C) on day 7 of osteogenic induction in mMSCs as in (A). (D) Measurement of ALP activity was performed in mMSCs as in (A). (E, F) The amount of Ca^{2+} (E) and citrate (F) deposited in differentiated mMSCs as in (A). $n = 4$. *$P < 0.05$ and **$P < 0.01$.

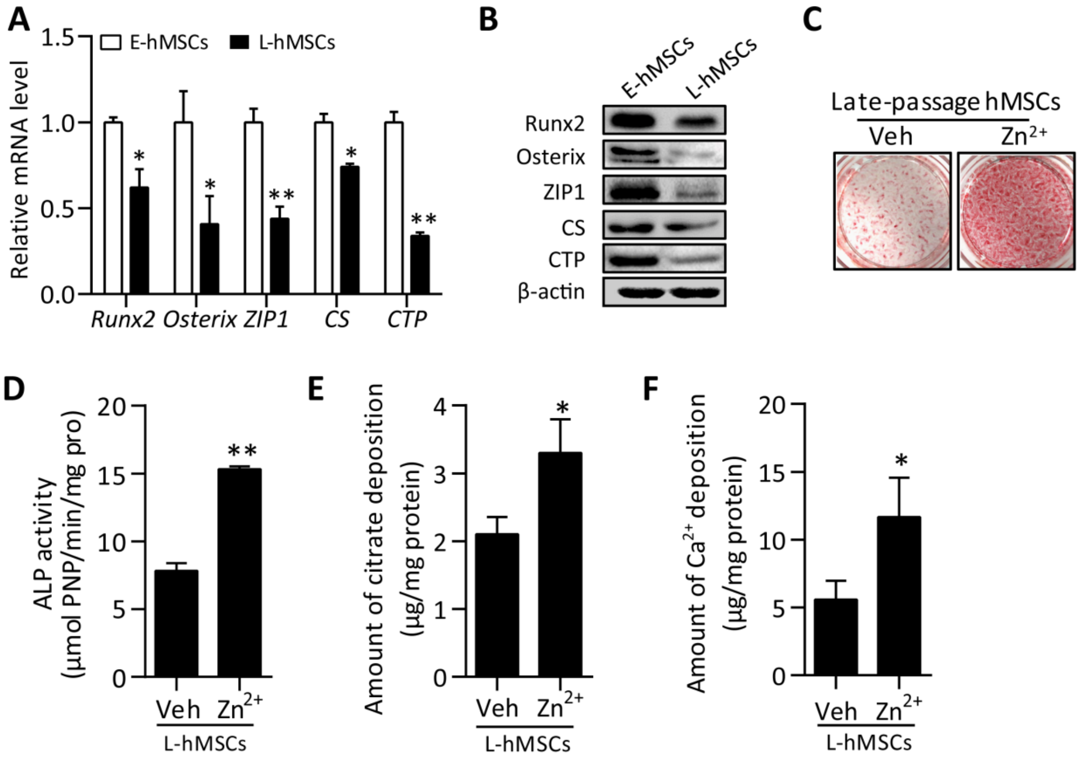

Figure S5. Zn^{2+} supplement enhanced osteogenic capacity and citrate deposition of late passage hMSCs. (A) qPCR analysis of indicated genes on day 14 of osteogenic induction in the Early-passage (E-, passage 6) and late-passage (L-, passage 16) hMSCs. (B) Western blot analysis of indicated proteins in differentiated hMSCs as (A). (C, D) Alizarin red S staining (C) and measurement of ALP activity (D) was performed in late-passage hMSCs treated with 20μM $ZnCl_2$ (Zn^{2+}) or vehicle (Veh) upon osteogenic induction. (E, F) The amount of citrate (E) and Ca^{2+} (F) deposition in differentiated hMSCs. $n = 4$. * $P < 0.05$ and ** $P < 0.01$.
